# An *Ipomoea batatas* Iron-Sulfur Cluster Scaffold Protein Gene, *IbNFU1*, Is Involved in Salt Tolerance

**DOI:** 10.1371/journal.pone.0093935

**Published:** 2014-04-02

**Authors:** Degao Liu, Lianjun Wang, Chenglong Liu, Xuejin Song, Shaozhen He, Hong Zhai, Qingchang Liu

**Affiliations:** 1 Beijing Key Laboratory of Crop Genetic Improvement/Laboratory of Crop Heterosis and Utilization, Ministry of Education, China Agricultural University, Beijing, China; 2 Institute of Food Crops, Hubei Academy of Agricultural Sciences, Wuhan, China; National Key Laboratory of Crop Genetic Improvement, China

## Abstract

Iron-sulfur cluster biosynthesis involving the nitrogen fixation (Nif) proteins has been proposed as a general mechanism acting in various organisms. NifU-like protein may play an important role in protecting plants against abiotic and biotic stresses. An iron-sulfur cluster scaffold protein gene, *IbNFU1*, was isolated from a salt-tolerant sweetpotato (*Ipomoea batatas* (L.) Lam.) line LM79 in our previous study, but its role in sweetpotato stress tolerance was not investigated. In the present study, the *IbNFU1* gene was introduced into a salt-sensitive sweetpotato cv. Lizixiang to characterize its function in salt tolerance. The *IbNFU1*-overexpressing sweetpotato plants exhibited significantly higher salt tolerance compared with the wild-type. Proline and reduced ascorbate content were significantly increased, whereas malonaldehyde (MDA) content was significantly decreased in the transgenic plants. The activities of superoxide dismutase (SOD) and photosynthesis were significantly enhanced in the transgenic plants. H_2_O_2_ was also found to be significantly less accumulated in the transgenic plants than in the wild-type. Overexpression of *IbNFU1* up-regulated pyrroline-5-carboxylate synthase (P5CS) and pyrroline-5-carboxylate reductase (P5CR) genes under salt stress. The systemic up-regulation of reactive oxygen species (ROS) scavenging genes was found in the transgenic plants under salt stress. These findings suggest that *IbNFU1*gene is involved in sweetpotato salt tolerance and enhances salt tolerance of the transgenic sweetpotato plants by regulating osmotic balance, protecting membrane integrity and photosynthesis and activating ROS scavenging system.

## Introduction

Soil salinization is becoming a serious threat to world agriculture to support a rapidly growing population [1], [2]. Approximately 20% of the irrigated soils in the world are under salt stress, and soil salinization has become a major constraint limiting crop production [3], [4]. The development of crops with elevated levels of salt tolerance is therefore highly desirable.

Iron–sulfur (Fe-S) clusters are cofactors of proteins that function in vital processes such as respiration, photosynthesis, sulfur and nitrogen assimilation, amino acid and purine metabolism, plant hormone and coenzyme synthesis, DNA repair and translation [5]. Ferredoxins are small, soluble [2Fe-2S] proteins that play a key role in electron distribution in all types of plastids [6], [7]. In addition, ferredoxin is a key regulator of ferredoxin-thioredoxin reductase in thioredoxin systems, and also contributes directly to antioxidant protection by its involvement in ascorbate and peroxiredoxin regeneration [8], [9], [10].

The biosynthesis of Fe-S clusters is a highly regulated process involving several proteins. Among them, so-called scaffold proteins play pivotal roles in both the assembly and delivery of Fe-S clusters. The Fe-S cluster scaffold protein involving the nitrogen fixation (Nif) was originally identified as a protein involved in the assembly of nitrogenase in a nitrogen-fixing bacterium, *Azotobacter vinelandii* [11], [12]. Later, NifU was shown to provide a scaffold for NifS-mediated assembly of Fe-S clusters [13], [14]. NFU proteins possess a conserved Cys-X-X-Cys motif in *Arabidopsi*s [15]. NFU2 is able to bind a [2Fe–2S] cluster that can subsequently be transferred to apo-ferredoxin and has a scaffold function for [4Fe-4S] and [2Fe-2S] ferredoxin cluster assembly [16], [17]. In cyanobacteria, knock-out mutants of *nfu* could not be obtained, indicating that this gene is essential [18]. The rice OsNifU1A domain II associates with ferredoxin to facilitate the efficient transfer of the Fe-S cluster from domain I to ferredoxin [19]. NifU-like protein gene was up-regulated when exposed to high salinity in *Saccharomyces cerevisiae*, drought in wheat and fungal stresses in wild rice (*Oryza minuta*) [20], [21], [22].

Sweetpotato, *Ipomoea batatas* (L.) Lam., is an important food and industrial material crop. It is also an alternative source of bio-energy as a raw material for fuel production [23]. The increased production of sweetpotato is desired, but this goal is often limited by salt stress [24]. Especially, sweetpotato as source of bio-energy will mainly be planted on marginal land. Salt stress is a critical delimiter for the cultivation expansion of sweetpotato. Therefore, the primary challenge facing scientists is enhancing sweetpotato's tolerance to salt stress to maintain productivity on marginal land. The improvement of this crop by conventional hybridization is limited because of its high male sterility, incompatibility and hexaploid nature [25]. Genetic engineering offers great potential to improve salt tolerance in this crop.

It is necessary to explore salt tolerance-associated genes in sweetpotato. In our previous study, the *IbNFU1* gene was isolated from a salt-tolerant sweetpotato line LM79 and the *IbNFU1*-overexpressing tobacco plants exhibited improved salt tolerance [26]. However, the role of *IbNFU1* in sweetpotato salt tolerance has not been investigated. Therefore, it is important to characterize the function of *IbNFU1* gene in sweetpotato. In the present study, we developed the *IbNFU1*-overexpressing sweetpotato plants and found that the *IbNFU1* gene is involved in sweetpotato salt tolerance.

## Materials and Methods

### Plant materials

Salt-sensitive sweetpotato cv. Lizixiang was employed in this study. Embryogenic suspension cultures of Lizixiang were prepared according to the method of Liu et al. [27]. Sixteen weeks after initiation, cell aggregates 0.7–1.3 mm in size from embryogenic suspension cultures of 3 days after subculture were employed for the transformation.

### Bacterial strain and plasmid

The *Agrobacterium tumefaciens* strain EHA105 harboring a binary vector, plasmid pCAMBIA1301, was used in this study. This binary vector contains the *IbNFU1* gene under the control of CaMV 35S promoter and NOS terminator of the expression box [26]. This vector also contained *gusA* and *hpt*IIgenes driven by a CaMV 35S promoter, respectively. The recombinant vector was transformed into the *A. tumefaciens* strain EHA 105 for sweetpotato transformation.

### Transformation and plant regeneration

The *Agrobacterium* suspension was prepared for the inoculation as described by Yu et al. [28]. Cell aggregates were infected for 5 min in the bacteria at room temperature, blotted on sterile filter paper, and then placed on filter paper in a Petri dish containing 25 mL solid Murashige and Skoog (MS) medium with 2.0 mg L^−1^ 2,4-dichlorophenoxyacetic acid (2,4-D) and 30 mg L^−1^ acetosyringone (AS) for the cocultivation. The cocultivation was conducted for 3 days in the dark at 27±1°C. After the cocultivation, the cell aggregates were washed twice with liquid MS medium containing 2.0 mg L^−1^ 2,4-D and 500 mg L^−1^ carbenicillin (Carb) and maintained for 1 week in liquid MS medium with 2.0 mg L^−1^ 2,4-D and 100 mg L^−1^ Carb on a reciprocal shaker (100 rpm) at 27±1°C under 13 h of cool-white fluorescent light at 10 μM m^−2^ s^−1^, and then were cultured at 2-week intervals on solid MS medium supplemented with 2.0 mg L^−1^ 2,4-D, 100 mg L^−1^ Carb and 25 mg L^−1^ hygromycin (Hyg) for the selection culture in the dark at 27±1°C. Eight weeks after selection, the obtained Hyg-resistant embryogenic calluses were transferred to solid MS medium with 1.0 mg L^−1^ abscisic acid (ABA), 100 mg L^−1^ Carb and 25 mg L^−1^ Hyg to induce formation of somatic embryos and regeneration of plantlets at 27±1°C under 13 h of cool-white fluorescent light at 54 μM m^−2^ s^−1^. The regenerated plantlets were further transferred to the basal medium for the development of whole plants under the same conditions.

### GUS assay and PCR analysis

The putatively transgenic plants were tested for GUS expression using histochemical GUS assay as described by Jefferson et al. [29]. The leaves, stems and roots of the putatively transgenic plants and wild-type plants were incubated for 12 h in GUS assay buffer at 37°C. Blue staining of the tissues denoted positive reaction.

Genomic DNA was extracted from the leaves of putatively transgenic plants and wild-type plants according to the instructions of EasyPure Plant Genomic DNA Kit (Transgen Biotech, Beijing, China). Equal amounts of 200 ng of total DNA were amplified in 50 μL reactions using 35S forward and *IbNFU1*-specific reverse primers (Table 1). These primers were expected to give products of 690 bp. PCR amplifications were performed with an initial denaturation at 94°C for 3 min, followed by 35 cycles at 94°C for 30 s, 55°C for 30 s, 72°C for 1 min and final extension at 72°C for 10 min. PCR products were separated by electrophoresis on a 1.0% (w/v) agarose gel.

**Table 1:** Primers used in this study.

Primer name	Primer sequence (5′-3′)
Primers for PCR	
35S-F	GAACTCGCCGTAAAGACTGG
*IbNFU1*-R	GGGGATTTCAACGAAGTGAA
Primers for Southern blot	
*hpt*II-F	ACAGCGTCTCCGACCTGATGCA
*hpt*II-R	AGTCAATGACCGCTGTTATGCG
Primers for real-time quantitative PCR	
*Actin*-F	AGCAGCATGAAGATTAAGGTTGTAGCAC
*Actin*-R	TGGAAAATTAGAAGCACTTCCTGTGAAC
*APX*-F	CCTGCTGGTCATTTACGTGA
*APX*-R	CTGGCCCATCTTTGGTGTAT
*DHAR*-F	TGTGTCAAGGCTGCTACTGG
*DHAR*-R	TTGCCTTCAGGAACCATTCA
*GPX*-F	GAACAGGGAAGGAAAGGTTG
*GPX*-R	TCTGAAACTTGGTGCTTCCA
*MDHAR*-F	CTACTCCCGTGCCTTTGATT
*MDHA*R-R	CTCCAAGAATGCACCAACAA
*NFU1*-F	TCGCAAGTACCCTCTGCTTT
*NFU1*-R	ATAGGGCCTTAGCCAGTGGT
*P5CR*-F	ATAGAGGCATTGGCTGATGG
*P5CR*-R	GGTAGTCCCACCTGGTGATG
*P5CS*-F	GCCTGATGCACTTGTTCAGA
*P5CS*-R	TTGAGCAATTCAGGGACCTC
*PRK*-F	GCTCTCAACATAGATCAGCT
*PRK*-R	TGAAGGCTCTACTATCTCAT
*psbA*-F	CATCCGTTGATGAATGGTTA
*psbA*-R	GCAACAGGAGCTGAGTATGC
*SOD*-F	TCCTGGACCTCATGGATTTC
*SOD*-R	GCCACTATGTTTCCCAGGTC

### In vitro assay for salt tolerance

Based on the method of He et al. [1], the transgenic plants and wild-type plants were cultured on MS medium with 86 mM NaCl in order to evaluate their in vitro salt tolerance at 27±1°C under 13 h of cool-white fluorescent light at 54 μM m^−2^ s^−1^. Three plants were treated for each line. The growth and rooting ability were continuously observed for 4 weeks.

### Analyses of proline and MDA content and SOD activity

Proline content and superoxide dismutase (SOD) activity were analyzed as described by He et al. [1]. Malonaldehyde (MDA) content was measured according to the method of Gao et al. [2].

### In vivo assay for salt tolerance

The transgenic plants and wild-type plants were transferred to soil in a greenhouse for further evaluation of salt tolerance. The cuttings about 25 cm in length were cultured in the Hoagland solution [30] with 0 and 86 mM NaCl, respectively. Three cuttings were treated for each line. The growth and rooting ability were continuously observed for 4 weeks.

The 25-cm-long cuttings of the salt-tolerant transgenic plants evaluated with water culture assay and wild-type plants were grown in 19-cm diameter pots containing a mixture of soil, vermiculite and humus (1∶1∶1, v/v/v) in a greenhouse, with one cutting per pot. All pots were irrigated sufficiently with half-Hoagland solution for 10 days until the cuttings formed new leaves. Each pot was then irrigated with a 200 mL of 200 mM NaCl solution once every 2 days for 2 weeks according to the method of Liu et al. [24]. After treatment, the plant fresh weight (FW) was measured immediately. The plants were then dried for 24 h in an oven at 80°C and weighed (DW). All treatments were performed in triplicate.

### Southern blot analysis

Genomic DNA was extracted from the leaves of the salt-tolerant transgenic plants and wild-type plants by cetyltrimethylammonium bromide (CTAB) method [31]. Approximately 20 μg genomic DNA of each sample was digested by *Hind* III. The restriction fragments were size-fractionated by 1.0% (w/v) agarose gel electrophoresis and transferred to a Hybond-N+ nylon membrane (Amersham Pharmacia Biotech, UK). The blot was hybridized with the DIG-labeled 591 bp *hpt*II probe and exposed to X-ray film for signal detection. The *hpt*II probe was obtained by PCR using the specific primers designed from the *hpt*II coding region (Table 1). PCR program conditions were as follows: 3 min at 94°C; 35 cycles of 30 s at 94°C, 30 s at 55°C and 60 s at 72°C, and followed by 10 min at 72°C. DNA probe preparation, hybridization and membrane washing were performed using DIG High Prime DNA Labeling and Detection Starter Kit II (Roche, Grenzacherstrasse, Basel, Switzerland).

### Measurement of photosynthesis

Photosynthetic rate, stomatal conductance and transpiration rate in the leaves of the salt-tolerant transgenic plants and wild-type plants grown in pots for 10 days under 200 mM NaCl stress were measured according to the methods of Liu et al. [24]. Relative chlorophyll content (SPAD value in fresh leaves) was measured as described by Fernández-Falcón et al. [32] with Chlorophyll Meter SPAD-502 (Minolta, Japan). The experiments were conducted at 9–11 a.m. of sunny days.

### Analysis of H_2_O_2_ accumulation

H_2_O_2_ accumulation in the leaves of the salt-tolerant transgenic plants and wild-type plants grown in pots for 10 days under 200 mM NaCl stress was analyzed by using 3, 3′-diaminobenzidine (DAB) staining as described by Liu et al. [24].

### Analysis of ascorbate content

Total ascorbate (reduced ascorbate plus oxidized ascorbate) and reduced ascorbate content in the leaves of the salt-tolerant transgenic plants and wild-type plants grown in pots for 10 days under 200 mM NaCl stress was analyzed according to the method reported by Lin et al. [33]. About 0.5 g of fully-expanded leaves per sample were ground into powder in liquid nitrogen with pre-chilled mortar and pestle, and mixed with 1 mL of 6% trichloroacetic acid (TCA). After centrifugation for 15 min at 18000×g at 4°C, the supernatant was transferred to a new centrifuge tube. For total ascorbate measurement, 100 μL supernatant was mixed with 50 μL of 100 mM dithiothreitol and 50 μL of 75 mM phosphate buffer (pH 7.0). The mixture was incubated for 30 min at 25°C and then reacted with a reaction buffer (250 μL 10% TCA, 200 μL 43% phosphoric acid, 200 μL 4% 2,2′-dipyridyl and 100 μL FeCl_3_) for 1 h at 37°C. The ascorbate concentration was determined by the absorbance at 525 nm according to the standard curves which were made using ascorbate standards (Sigma, St. Louis, MO, USA) in 6% TCA. For reduced ascorbate determination, 100 μL supernatant was added with 50 μL of deionized water and 50 μL of 75 mM phosphate buffer (pH 7.0) and incubated for 30 min at 25°C, then reduced ascorbate was measured as mentioned above.

### Expression analyses of proline biosynthesis, photosynthesis and ROS scavenging genes

The expression of genes related to proline biosynthesis, photosynthesis and ROS scavenging in the salt-tolerant transgenic plants and wild-type plants was analyzed by real-time quantitative PCR (qRT-PCR). The transgenic and wild-type in vitro-grown plants were submerged in 1/2 MS medium containing 200 mM NaCl and sampled at 0, 3, 6, 12, 24 and 48 h after treatment. The qRT-PCR analysis was performed as described by Liu et al. [24]. Specific primers designed from conserved regions of genes were listed in Table 1. Sweetpotato *β-actin* gene (accession No. AY905538) was used as an internal control (Table 1). Quantification of the gene expression was done with comparative *C*_T_ method [34].

### Statistical analysis

The experiments were repeated three times and the data presented as the mean ± SE were analyzed by Student's *t*-test in a two-tailed analysis to compare the parameters obtained under normal or salt stress conditions. A *P* value of <0.05 or <0.01 was considered to be statistically significant.

## Results

### Production of the *IbNFU1-*overexpressing sweetpotato plants

**Figure 1:**
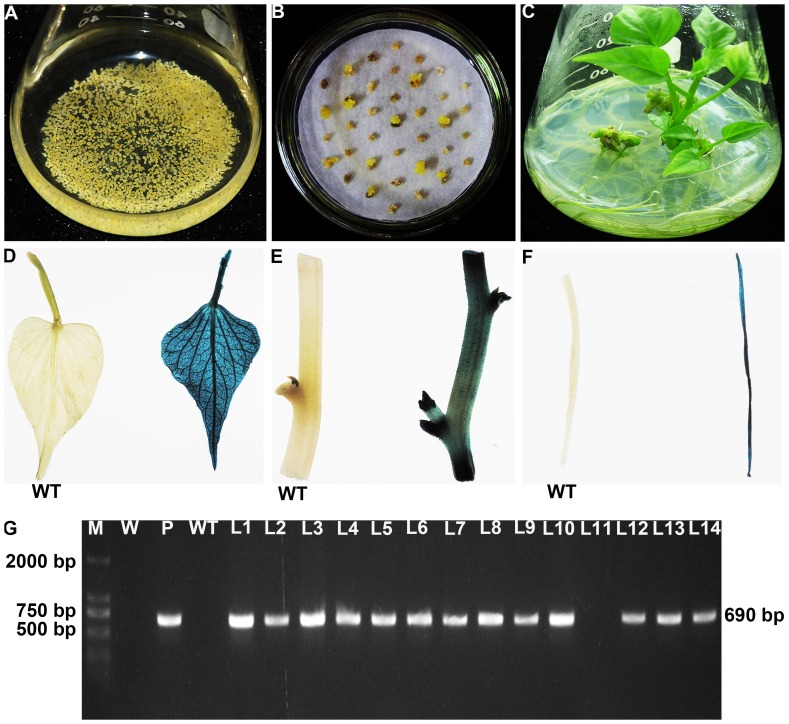
Production of transgenic sweetpotato plants overexpressing the *IbNFU1* gene. (A) Embryogenic suspension cultures rapidly proliferating in MS medium containing 2.0 mg L^−1^ 2,4-D. (B) Hyg-resistant calluses formed on MS medium with 2.0 mg L^−1^ 2,4-D, 100 mg L^−1^ Carb and 25 mg L^−1^ Hyg after 8 weeks of selection. (C) Regeneration of plantlets from Hyg-resistant calluses on MS medium with 1.0 mg L^−1^ ABA, 100 mg L^−1^ Carb and 25 mg L^−1^ Hyg. (D), (E) and (F) GUS expression in leaf, stem and root of a transgenic plant and no GUS expression in the wild-type (WT). (G) PCR analysis of transgenic plants. Lane M: DL2000 DNA marker; Lane W: water as negative control; Lane P: plasmid pCAMBIA1301-*IbNFU1* as positive control; Lane WT: wild-type as negative control; Lanes L1–L10, L12–L14: transgenic plants; Lane L11: non-transgenic plants.

A total of 1000 cell aggregates of sweetpotato cv. Lizixiang (Figure 1A) cocultivated with *A. tumefaciens* strain EHA 105 were cultured on the selective medium with 2.0 mg L^−1^ 2,4-D, 100 mg L^−1^ Carb and 25 mg L^−1^ Hyg. Eight weeks after selection, 35 Hyg-resistant embryogenic calluses were produced from them (Figure 1B) and transferred to MS medium with 1.0 mg L^−1^ ABA, 100 mg L^−1^ Carb and 25 mg L^−1^ Hyg. After 5 to 6 weeks, 28 of them formed somatic embryos which further germinated into plantlets on the same medium (Figure 1C). These plantlets developed into whole plants on the basal medium. A total of 42 putatively transgenic plants, named L1, L2, …, L42, respectively, were obtained in the present study.

The 42 putatively transgenic plants were analyzed by GUS assay. The results showed that 36 of them had visible GUS activity in leaf, stem and root tissues, indicating stable *gusA* gene integration into the genome of the plants (Figure 1D, E, F). The remaining 6 plants and wild-type plants showed no GUS expression (Figure 1D, E, F). PCR analysis revealed that all of the 36 GUS-positive plants had a specific 690 bp band of the *IbNFU1* gene, while no specific band was observed in the 6 GUS-negative plants, wild-type plants and water (Figure 1G), indicating that the 36 plants were transgenic.

### Improved salt tolerance in the *IbNFU1*-overexpressing sweetpotato

**Figure 2:**
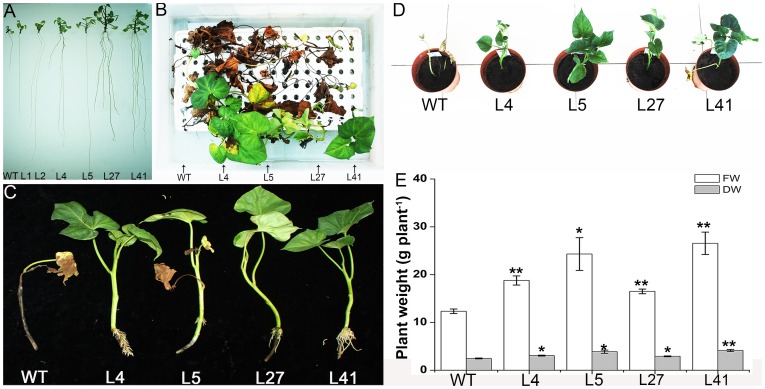
Responses of the *IbNFU1*-overexpressing sweetpotato plants under salt stress. (A) The growth and rooting of transgenic plants (L1, L2, L4, L5, L27 and L41) and wild-type plant (WT) cultured for 4 weeks on MS medium supplemented with 86 mM NaCl. (B) and (C) Phenotypes of salt-tolerant transgenic plants (L4, L5, L27 and L41) and WT incubated for 4 weeks in Hoagland solution with 86 mM NaCl. (D) and (E) Phenotypes, fresh weight (FW) and dry weight (DW) of transgenic plants and WT grown in pots under 200 mM NaCl stress. A plant grown in a 19-cm diameter pot was irrigated with a 200 mL of 200 mM NaCl solution once every 2 days for 2 weeks. Data are presented as means ± SE (n = 3). * and ** indicate a significant difference from that of WT at *P*<0.05 and <0.01, respectively, by Student's *t*-test.

Thirty-six transgenic plants and wild-type plants were cultured on MS medium with 86 mM NaCl for 4 weeks. The transgenic plants exhibited vigorous growth and good rooting in contrast to the poor-growing wild-type plants (Figure 2A). This observation indicated that the transgenic plants had higher salt tolerance than wild-type plants.

Proline and MDA content and SOD activity of the 36 transgenic plants were shown in Table 2. Proline content and SOD activity were significantly higher in the 13 transgenic pants than in wild-type plants, while MDA content was significantly lower in these 13 transgenic plants than in wild-type plants. These results suggest that the high salt tolerance observed is due, at least in part, to the modulation of existing salt tolerance pathways.

**Table 2:** Comparison of salt tolerance between the *IbNFU1*-overexpressing sweetpotato plants and wild-type plants.

Plant lines	Proline content (μg g^−1^ FW)	SOD activity (U g^−1^ FW)	MDA content (nM g^−1^ FW)
L41	66.51±1.12^**^ a	553.51±2.63^**^	9.42±0.16^**^
L4	62.85±1.38^**^	526.37±3.67^**^	11.72±0.16^**^
L5	60.63±2.55^**^	488.20±3.12^**^	8.84±0.12^**^
L27	59.93±1.52^**^	540.53±0.78^**^	6.90±0.23^**^
L24	59.02±2.47^**^	447.09±2.76^**^	9.87±0.31^**^
L32	58.27±1.89^**^	369.33±5.90^**^	12.82±0.06^**^
L15	58.21±1.17^**^	382.57±7.55^**^	13.87±0.27
L35	58.21±2.39^**^	375.87±2.13^**^	14.76±0.34
L6	57.99±1.08_**_	368.88±1.36^**^	12.37±0.25^**^
L19	55.37±1.09^**^	429.53±2.66^**^	13.16±0.14^**^
L40	54.56±1.85^**^	357.45±6.44^*^	13.72±0.28
L39	53.69±1.46^**^	361.69±4.56^**^	13.10±0.05^**^
L16	53.24±0.84^**^	362.51±0.43^**^	13.31±0.19^**^
L13	52.80±0.47^**^	368.52±4.48^**^	13.73±0.14^*^
L12	51.87±1.26^**^	444.20±5.51^**^	13.60±0.07^**^
L2	50.26±1.07^**^	356.73±5.13^**^	14.16±0.18
L37	50.10±1.79^**^	358.82±3.21^**^	14.04±0.13
L26	48.47±1.57^**^	343.32±3.67^*^	14.66±0.12
L1	48.35±2.29^*^	345.14±1.27^*^	14.25±0.06
L33	46.71±2.84^*^	320.68±9.48	13.84±0.28
L25	46.45±2.92^*^	416.38±4.49^**^	13.41±0.06^**^
L9	46.34±2.04^*^	336.25±3.30	13.38±0.24^*^
L31	44.23±1.01^*^	362.74±5.9^**^	14.05±0.07
L34	43.55±2.02^*^	340.87±1.24^*^	14.55±0.11
L3	41.13±2.63	320.98±2.26	14.94±0.16
L28	40.31±2.07	336.11±8.78	14.72±0.05
L42	40.10±1.77	361.08±7.53^*^	12.82±0.25^**^
L7	40.10±1.16	326.88±7.43	14.51±0.10
L38	39.30±0.58	352.81±0.50^**^	13.79±0.08^*^
L8	39.30±0.58	328.74±5.07	14.58±0.02
L14	39.18±1.18	353.53±5.77^*^	14.09±0.07
L36	38.20±2.19	361.13±7.06^*^	13.64±0.18^*^
L10	36.78±2.16	319.00±4.23	16.57±0.19
WT	34.43±2.43	320.20±5.67	15.06±0.21
L20	34.16±1.03	303.57±0.11	13.85±0.28
L29	33.78±1.18	291.48±3.11	17.33±0.27
L17	33.62±2.07	290.13±2.32	16.89±0.26

a Data are presented as means ± SE (n = 3). * and ** indicate a significant difference from that of the wild-type (WT) at *P*<0.05 and <0.01, respectively, by Student's *t*-test.

The 13 transgenic plants and wild-type plants were transferred to the soil in a greenhouse and showed 100% survival. No morphological variations were observed. For further evaluation of salt tolerance, the cuttings of these 13 transgenic plants and wild-type plants were cultured for 4 weeks in the Hoagland solution containing 0 and 86 mM NaCl, respectively. The growth and rooting of all cuttings were normal without NaCl. And at 86 mM NaCl, the 4 transgenic plants (L4, L5, L27 and L41) formed obvious new leaves and roots; the 4 transgenic plants survived, but failed to form new leaves; the 5 transgenic plants and wild-type plants gradually turned brown to death (Figure 2B, C; Table 3). These results demonstrated that L4, L5, L27 and L41 had significantly higher salt tolerance than the other transgenic plants and wild-type plants.

**Table 3:** Leaf and root formation of the *IbNFU1*-overexpressing sweetpotato plants after 4 weeks of water culture with 86 mM NaCl.

Plant lines	Leaf formation	No. of roots
L41	++^a^	22.67±4.70^**^ b
L4	++	21.33±4.33^**^
L5	++	13.00±3.06^*^
L27	++	11.67±3.18^*^
L32	+	8.67±2.73^*^
L24	+	8.33±2.60^*^
L39	+	7.67±3.71
L19	+	7.00±2.31
L6	−	5.67±2.03
L12	−	5.00±2.08
L16	−	4.67±1.76
L13	−	4.33±1.45
L25	−	2.00±1.53
WT	−	0.67±0.33

a ‘++’ indicates that cuttings formed obvious new leaves; ‘+’ indicates that cuttings survived, but failed to form new leaves; ‘−’ indicates that cuttings died;

b Data are presented as means ± SE (n = 3). * and ** indicate a significant difference from that of the wild-type (WT) at *P*<0.05 and <0.01, respectively, by Student's *t*-test.

The 4 salt-tolerant transgenic plants (L4, L5, L27 and L41) and wild-type plants were grown in pots and irrigated with a 200 mL of 200 mM NaCl solution once every 2 days for 2 weeks. The 4 salt-tolerant plants showed good growth and increased physical size, while wild-type plants died (Figure 2D). FW and DW of the 4 salt-tolerant plants were increased by 34–115% and 18–67%, respectively, compared to the wild-type (Figure 2E).

### Southern blot analysis of the salt-tolerant transgenic plants

**Figure 3:**
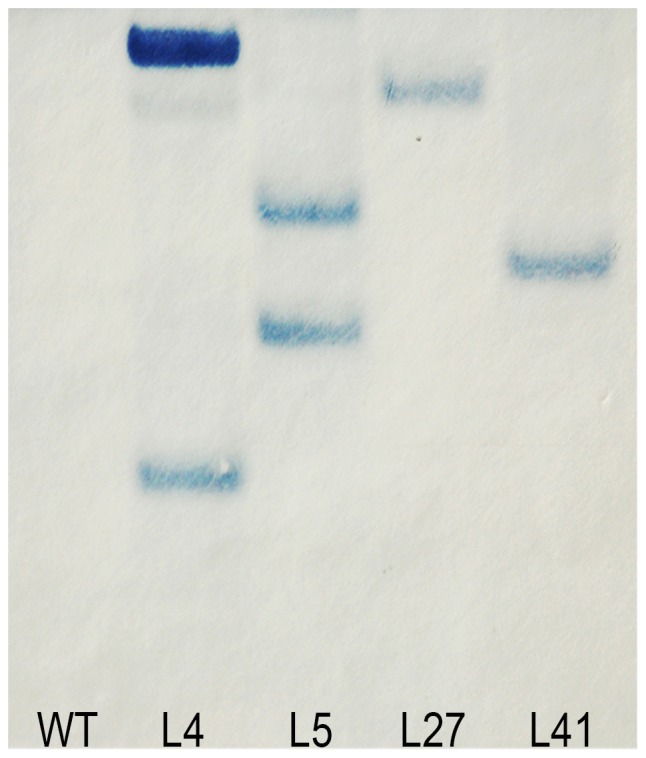
Southern blot analysis of the salt-tolerant sweetpotato plants overexpressing the *IbNFU1* gene. DNA was digested with *Hind* III and hybridized with the DIG-labeled *hpt* II gene probe. Hybridizaton signals revealed were indication of copy numbers of transgene insertion. Lane WT: wild-type plant; Lanes L4, 5, L27 and L41: salt-tolerant transgenic plants.

Transgene integration patterns of the 4 salt-tolerant transgenic plants were analyzed by Southern bolt. The DNA of transgenic plants and wild-type plants was digested with *Hind* III, which has a unique cleavage site in the T-DNA region in the vector and hybridized with the *hpt*II gene probe. The transgenic plants displayed different patterns and the copy number of integrated gene varied from 1 to 2. No hybridizing band was observed in the wild-type plants as expected (Figure 3).

### qRT-PCR analysis of the salt-tolerant transgenic plants

**Figure 4:**
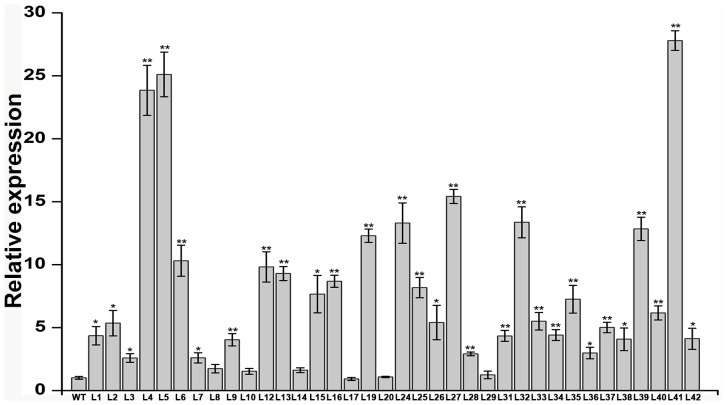
Expression analysis of *IbNFU1* gene in the transgenic sweetpotato plants by real-time quantitative PCR. The 36 transgenic and wild-type (WT) in vitro-grown plants were submerged in 1/2 MS medium containing 200 mM NaCl for 6 h to analyze the expression of *IbNFU1*. The sweetpotato *β-actin* gene was used as an internal control. The results are expressed as relative values based on wild-type plants as reference sample set to 1.0. Data are presented as means ± SE (n = 3). * and ** indicate a significant difference from that of WT at *P*<0.05 and <0.01, respectively, by Student's *t*-test.

The 36 transgenic plants were further analyzed by qRT-PCR. The results indicated that there was positive correlationship between expression level of *IbNFU1* gene and salt tolerance of transgenic plants (Figure 4). Significantly higher level of *IbNFU1* gene expression was found in the 4 salt-tolerant transgenic plants (L4, L5, L27 and L41) than in the other 32 transgenic plants and wild-type (Figure 4).

### Enhanced photosynthesis in the salt-tolerant transgenic plants

**Figure 5:**
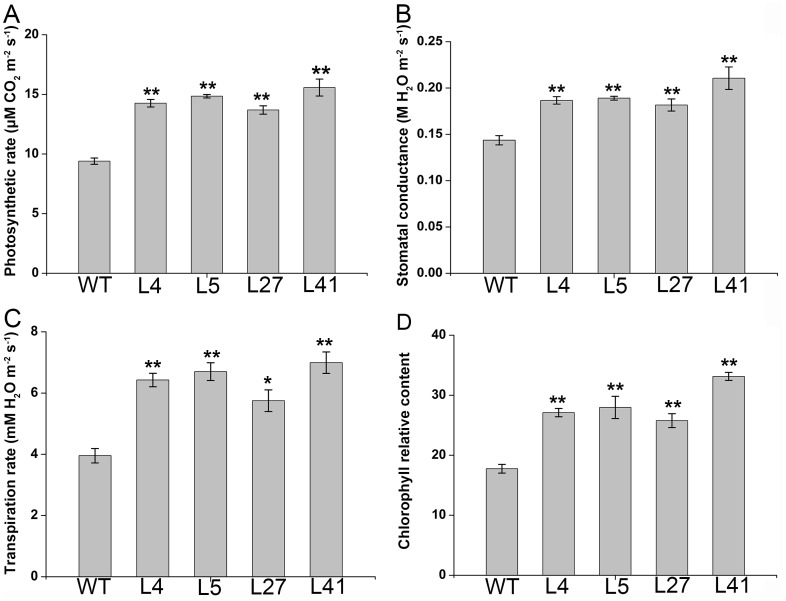
Photosynthetic performance of the *IbNFU1*-overexpressing sweetpotato plants under salt stress. (A), (B), (C) and (D) Photosynthetic rate, stomatal conductance, transpiration rate and chlorophyll relative content, respectively, in the leaves of salt-tolerant transgenic plants (L4, L5, L27 and L41) and wild-type plant (WT). The 25-cm-long cuttings of the salt-tolerant transgenic plants evaluated with water culture assay and WT were grown in 19-cm diameter pots containing a mixture of soil, vermiculite and humus (1∶1∶1, v/v/v) in a greenhouse, with one cutting per pot. All pots were irrigated sufficiently with half-Hoagland solution for 10 days until the cuttings formed new leaves, and then each pot was irrigated with a 200 mL of 200 mM NaCl solution once every 2 days for 10 days. Data are presented as means ± SE (n = 3). * and ** indicate a significant difference from that of WT at *P*<0.05 and <0.01, respectively, by Student's *t*-test.

Photosynthesis in the leaves of the 4 salt-tolerant transgenic plants grown in pots for 10 days under 200 mM NaCl stress was measured. The salt-tolerant transgenic plants maintained significantly higher photosynthetic rate, stomatal conductance, transpiration rate and chlorophyll relative content, which were increased by 46–66%, 26–47%, 45–77% and 45–87%, respectively, compared to the wild-type (Figure 5A, B, C, D).

### Reduced H_2_O_2_ accumulation in the salt-tolerant transgenic plants

**Figure 6:**
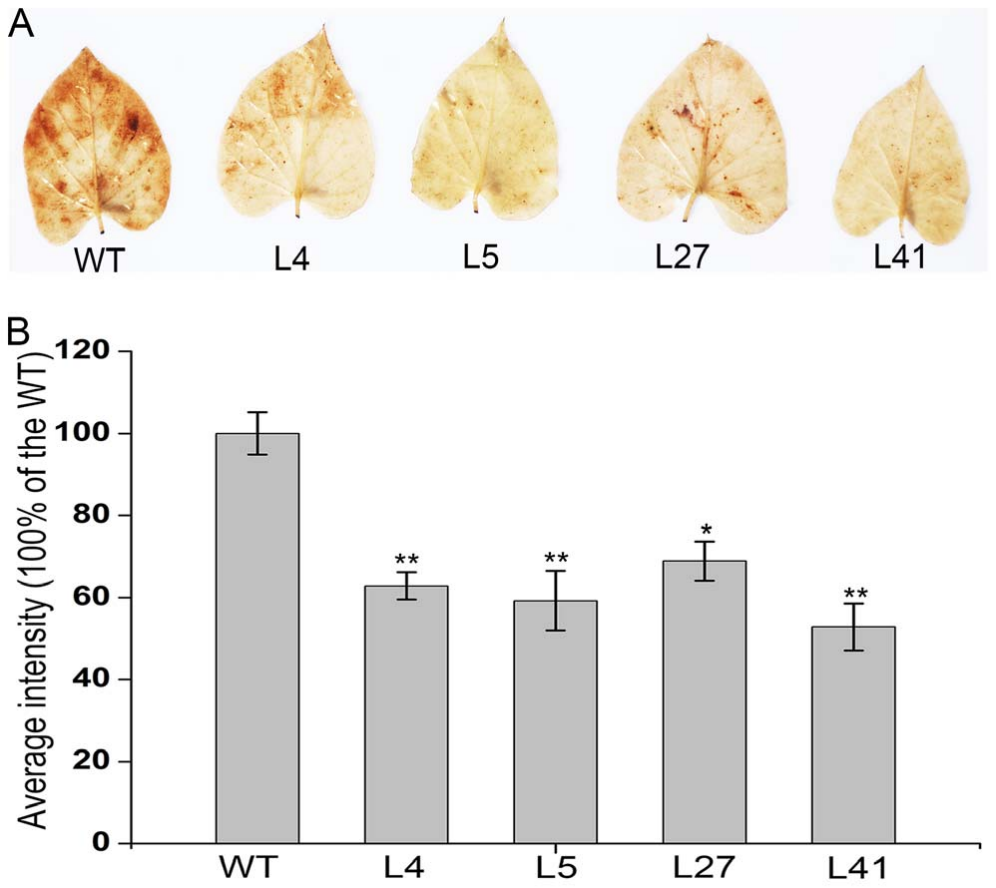
Effects of salt stress on H_2_O_2_ accumulation in the *IbNFU1*-overexpressing sweetpotato plants. (A) Accumulation of H_2_O_2_ in the leaves of salt-tolerant transgenic plants (L4, L5, L27 and L41) and wild-type plant (WT). A plant grown in a 19-cm diameter pot was irrigated with a 200 mL of 200 mM NaCl solution once every 2 days for 10 days. (B) The average intensity of DAB staining leaves after converting to 256 grey scale images. Data are presented as means ± SE (n = 3). * and ** indicate a significant difference from that of WT at *P*<0.05 and <0.01, respectively, by Student's *t*-test.

Abiotic stress induces the accumulation of H_2_O_2_, which is the toxic molecule that causes oxidative damage in plants [35]. To explore the potential mechanism by which *IbNFU1* improved salt tolerance in sweetpotato, H_2_O_2_ accumulation was analyzed by using DAB staining of leaves from the 4 salt-tolerant transgenic plants and wild-type plants under 200 mM NaCl stress for 10 days. The leaves of the salt-tolerant transgenic plants displayed less brown spots and diffuse staining than those of wild-type plants, indicating less H_2_O_2_ accumulation in the salt-tolerant transgenic plants (Figure 6A). The statistical analysis further confirmed that significantly less H_2_O_2_ was accumulated in the salt-tolerant transgenic plants compared to the wild-type under salt stress (Figure 6B).

### Increased reduced ascorbate level in the salt-tolerant transgenic plants

**Figure 7:**
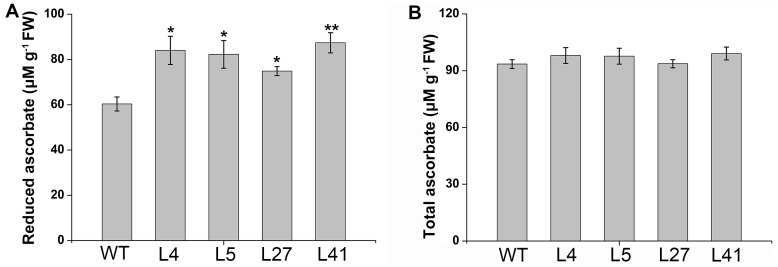
Ascorbate content of the *IbNFU1*-overexpressing sweetpotato plants under salt stress. (A) and (B) Reduced ascorbate content and total ascorbate content, respectively, in the leaves of salt-tolerant transgenic plants (L4, L5, L27 and L41) and wild-type plant (WT). A plant grown in a 19-cm diameter pot was irrigated with a 200 mL of 200 mM NaCl solution once every 2 days for 10 days. Data are presented as means ± SE (n = 3). * and ** indicate a significant difference from that of WT at *P*<0.05 and <0.01, respectively, by Student's *t*-test.

Reduced ascorbate is a major antioxidant reacting directly with hydroxyl radicals, superoxide anion, and singlet oxygen [36], [37]. To explore the potential mechanism by which *IbNFU1* reduced H_2_O_2_ accumulation in the salt-tolerant transgenic plants, total ascorbate and reduced ascorbate content was measured in leaves of the salt-tolerant transgenic plants and wild-type plants under 200 mM NaCl stress for 10 days. Total ascorbate content was not obviously changed, while reduced ascorbate content was significantly increased by 15–45% in the salt-tolerant transgenic plants compared to the wild-type (Figure 7).

### Expression analyses of proline biosynthesis, photosynthesis and ROS scavenging genes

**Figure 8:**
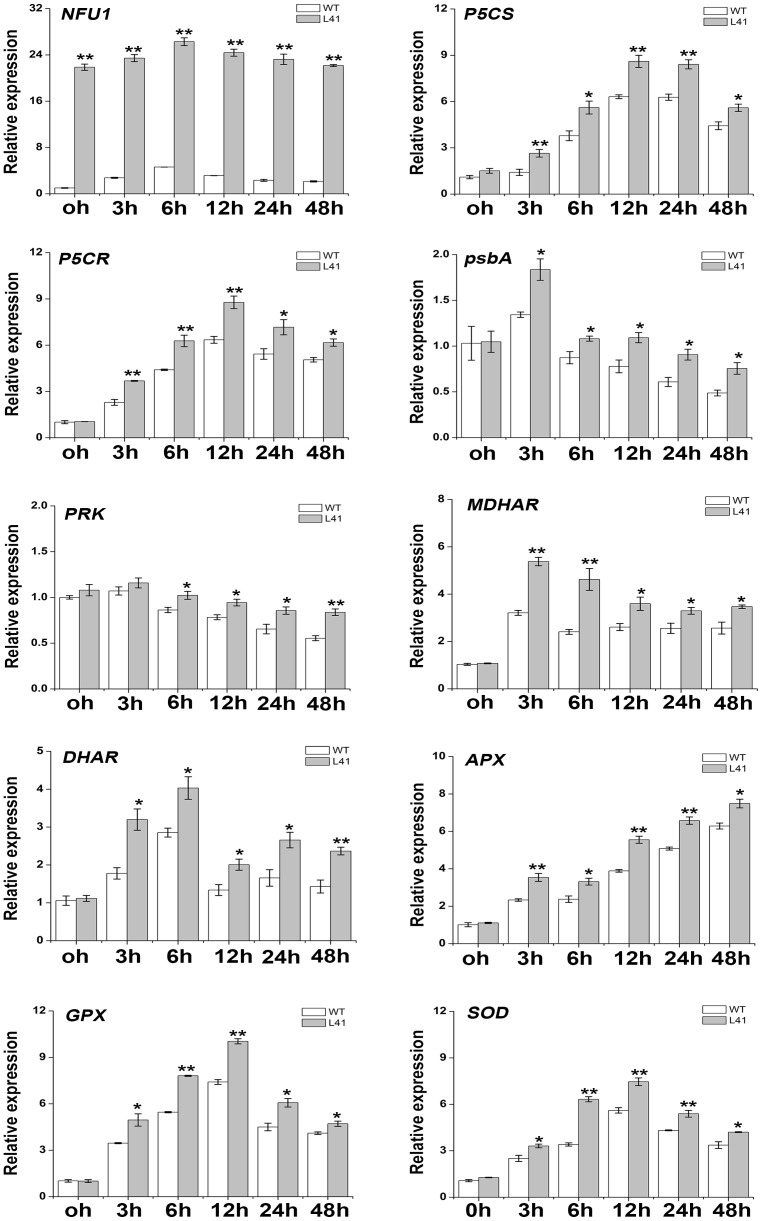
Relative expression level of *IbNFU1* and its related genes in the *IbNFU1*-overexpressing sweetpotato plants. *P5CS*: pyrroline-5-carboxylate synthase; *P5CR*: pyrroline-5-carboxylate reductase; *psbA*: encoding D1 protein; *PRK*: phosphoribulokinase (PRKase); *MDHAR*: monodehydroascorbate reductase; *DHAR*: dehydroascorbate reductase; *APX*: ascorbate peroxidase; *GPX*: glutathione peroxidase; *SOD*: superoxide dismutase. The transgenic (L41) and wild-type (WT) in vitro-grown plants were submerged in 1/2 MS medium containing 200 mM NaCl and sampled at 0, 3, 6, 12, 24 and 48 h after treatment to analyze the expression level of genes. The sweetpotato *β-actin* gene was used as an internal control. The results are expressed as relative values based on WT grown under control condition as reference sample set to 1.0. Data are presented as means ± SE (n = 3). * and ** indicate a significant difference from that of WT at *P*<0.05 and <0.01, respectively, by Student's *t*-test.

Expression of *IbNFU1*, proline biosynthesis, photosynthesis and ROS scavenging genes in the salt-tolerant transgenic plant L41 was analyzed by qRT-PCR. The expression level of *IbNFU1* gene was significantly higher in L41 compared to the wild-type without or with salt stress imposition at all time points (Figure 8). To investigate the impact of *IbNFU1* overexpression on the transcription of salt stress response pathways related genes, the expression of well-known salt stress responsive marker genes encoding pyrroline-5-carboxylate synthase (P5CS), pyrroline-5-carboxylate reductase (P5CR), monodehydroascorbate reductase (MDHAR), dehydroascorbate reductase (DHAR), ascorbate peroxidase (APX), glutathione peroxidase (GPX) and SOD was analyzed under salt stress (Figure 8). *P5CS*, *P5CR*, *MDHAR*, *DHAR*, *APX*, *GPX* and *SOD* genes exhibited significantly increased expression level in L41 compared to the wild-type at all time points under salt stress (Figure 8). The expression level of *psbA* and *PRK* genes, which encode D1 protein and phosphoribulokinase (PRKase), respectively, were significantly higher in L41 than in the wild-type.

## Discussion

Soil salinity is one of the major factors that limit the productivity and quality of crops. Plant genetic engineering provides the potential for breeding salt-tolerant varieties. Overexpression of salt tolerance related genes is an important strategy for improving salt tolerance of crops. A few salt tolerance-associated genes have been isolated from sweetpotato. Chen et al. [38] isolated *SPCP2* gene from sweetpotato and the *SPCP2*-overexpressing *Arabidopsis* plants exhibited higher salt and drought tolerance. Liu et al. [24] cloned *IbP5CR* gene from sweetpotato and the *IbP5CR*-overexpressing sweetpotato plants exhibited higher salt tolerance.

NifU-like protein gene was up-regulated when exposed to high salinity in *Saccharomyces cerevisiae*, drought in wheat and fungal stresses in wild rice (*Oryza minuta*) [20], [21], [22]. In our previous study, the *IbNFU1* gene was isolated from a salt-tolerant sweetpotato line LM79 and the *IbNFU1*-overexpressing tobacco plants exhibited improved salt tolerance [26]. In the present study, we produced the transgenic plants of the salt-sensitive sweetpotato cv. Lizixiang overexpressing the *IbNFU1* gene and found that overexpression of *IbNFU1* can significantly enhance the salt tolerance of sweetpotato (Figure 2). It is suggested that the *IbNFU1* gene plays an important role in response of sweetpotato to salt stress.

Osmotic stress often results in more accumulation of proline, and the level of proline accumulation is related to the extent of salt tolerance [24], [39]–[43]. In the present study, most of the *IbNFU1-*overexpressing sweetpotato plants had significantly higher proline content compared to wild-type plants under salt stress, indicating measurable improvement of salt tolerance (Table 2; Figure 2). Proline accumulation in the *IbNFU1-*overexpressing sweetpotato plants most likely maintains the osmotic balance between the intracellular and extracellular environment under salt stress, which results in the improved salt tolerance [44], [45], [46]. Also, proline helps cells to maintain membrane integrity [45], [47], [48] and has been proposed to function as molecular chaperone stabilizing the structure of proteins [49]. Therefore, it is assumed that proline accumulation in the *IbNFU1-*overexpressing sweetpotato plants might protect the cell membrane from salt-induced injuries.

**Figure 9:**
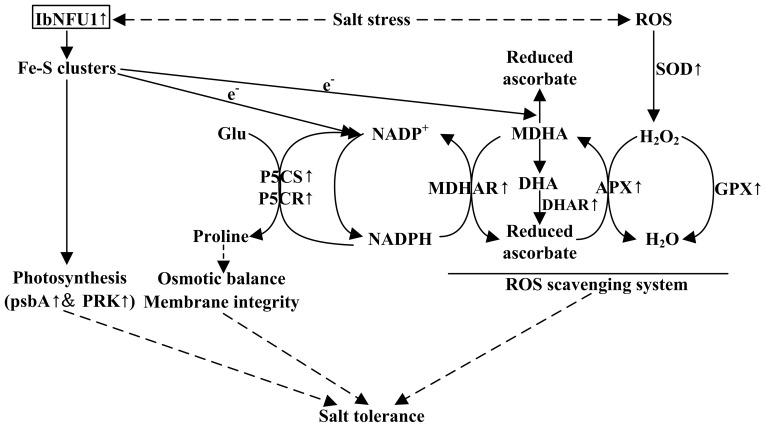
Diagram showing the regulation of proline accumulation, photosynthesis and ROS-scavenging system in the *IbNFU1*-overexpressing sweetpotato plants. ‘↑’ indicates up-regulation of expression of genes coding these enzymes. The *IbNFU1* gene plays a pivotal role in both assembly and delivery of Fe-S clusters. These Fe-S clusters are required for photosynthesis and donate eletrons in proline biosynthesis and ascorbate-mediated ROS scavenging system, resulting in more proline accumulation and ROS scavenging system activation by up-regulating *P5CS*, *P5CR* and ROS-scavenging genes under salt stress. Overexpression of *IbNFU1* enhances salt tolerance of the transgenic plants by regulating osmotic balance, protecting membrane integrity and photosynthesis and activating ROS scavenging system through the up-regulation of ROS-scavenging genes.

The *Arabidopsis* NifU-like protein NFU2 has an important function as a scaffold protein required for [4Fe-4S] and [2Fe-2S] ferredoxin cluster assembly [16], [17]. In rice, the OsNifU1A domain II associates with ferredoxin to facilitate the efficient transfer of the Fe-S cluster from domain I to ferredoxin [19]. Ferredoxins are small, soluble [2Fe-2S] proteins that play a key role in electron distribution in all types of plastids [6]. Electrons from reduced ferredoxins are accepted by ferredoxin-NADP^+^-oxidoreductase to generate NADPH [7]. Proline biosynthesis is a reductive pathway, and requires NADPH for the reduction of glutamate to pyrroline-5-carboxylate (P5C) by the P5CS enzyme and P5C to proline by P5CR to generate NADP^+^ that can be used further as electron acceptor [40], [50]–[53]. In the present study, the expression of *P5CS* and *P5CR* genes was up-regulated in the transgenic sweetpotato plants under salt stress (Figure 8). It is suggested that the up-regulated expression of *P5CS* and *P5CR* genes in the *IbNFU1-*overexpressing sweetpotato plants under salt stress is due to the increased ratio of NADPH/NADP^+^, which result in more accumulation of proline under salt stress (Figure 9).

MDA is often considered a reflection of cellular membrane degradation, and its accumulation increases with production of superoxide radicals and hydrogen peroxide [35]. Higher MDA content can induce cell membrane damage, which further reduces salt tolerance of plants [24], [54]–[57]. In the present study, most of the *IbNFU1-*overexpressing sweetpotato plants had significantly lower MDA content compared to wild-type plants, also indicating the marked improvement of their salt tolerance (Table 2).

Salinity perturbs plant water uptake in leaves, leading to quick response in stomatal conductance. It also disrupts the osmotic, ionic and nutrient balances in plants. This affects photosynthetic electron transport, NADPH formation and the activities of enzymes for carbon fixation [24], [58], [59], [60]. The main function of Fe–S proteins is electron transfer through the Fe^2+^ or Fe^3+^ oxidation states of iron. Fe–S proteins are keys to electron transfer in the respiratory complexes of mitochondria and in the photosynthetic apparatus of chloroplasts [61]. The *Arabidopsis* NifU-like proteins NFU2 is required for biogenesis of photosystem I [16], [17]. In our study, the *IbNFU1-*overexpressing sweetpotato plants exhibited higher photosynthetic rate, stomatal conductance, transpiration rate and chlorophyll relative content compared to wild-type plants under salt stress (Figure 5). Also, the expression of *psbA* and *PRK* genes was up-regulated in the transgenic plants (Figure 8). The biomass difference between the *IbNFU1*-overexpressing plants and wild-type plants might be due to the photosynthesis difference under salt stress (Figure 2E). The less affected photosynthesis of the *IbNFU1-*overexpressing sweetpotato plants could be explained by that *IbNFU1* have an important function as a molecular scaffold for Fe-S cluster biosynthesis and is required for biogenesis of photosystem I (Figure 9).

Salinity leads to the overproduction of ROS in plants which are highly reactive and toxic and cause damage to proteins, lipids, carbohydrates and DNA which ultimately results in oxidative stress. ROS scavenging systems of plants detoxify ROS to minimize and/or prevent oxidative damage in cells by increasing the activity of ROS scavenging enzymes [62]. As a key enzyme of ROS scavenging system, SOD is usually induced by salinity to enhance the timely dismutation of superoxide into oxygen and H_2_O_2_, which is subsequently removed through different pathways [63], [64]. Thus, SOD activity is often used to test the salt tolerance of plants [1], [2], [24], [63], [65], [66]. In the present study, most of the *IbNFU1-*overexpressing sweetpotato plants had significantly higher SOD activity compared to wild-type plants, which further showed the marked improvement of their salt tolerance (Table 2). The accumulation of H_2_O_2_ was significantly less in the *IbNFU1*-overexpressing sweetpotato plants than in wild-type plants under salt stress (Figure 6). Consistent with this phenomenon, the increased SOD expression and activity were detected in the transgenic plants (Table 2; Figure 8). In parallel, the expression of other important ROS-scavenging genes, including *MDHAR*, *DHAR*, *APX* and *GPX*, was systematically up-regulated at the transcriptional level (Figure 8), suggesting that the improved salt tolerance of the transgenic sweetpotato plants is also due to the enhanced ROS scavenging (Figure 9) [24], [64], [67], [68].

Reduced ascorbate is a major antioxidant reacting directly with hydroxyl radicals, superoxide anion, and singlet oxygen and can be recycled by several different mechanisms [36], [37]. The short-lived monodehydroascorbate (MDHA) radical, produced following reduced ascorbate oxidation, can be recycled following reduction by ferredoxin or MDHAR. MDHA can also undergo disproportionation into dehydroascorbate (DHA) and reduced ascorbate. DHA can be recycled into reduced ascorbate by DHAR before it undergoes irrevocable hydrolysis. The DHAR- and MDHAR-mediated mechanisms of ascorbate recycling are important in detoxifying ROS under salt stress [69]. Reduced ferredoxin and the generated NADPH can donate electrons to MDHA to generate reduced ascorbate, which is employed by APX to scavenge H_2_O_2_ [70]–[73]. In our study, the *IbNFU1-*overexpressing sweetpotato plants had significantly higher reduced ascorbate content compared to wild-type plants (Figure 7). Moreover, the ascorbate recycling related genes *MDHAR*, *DHAR* and *APX* were up-regulated in the *IbNFU1*-overexpressing plants than in wild-type plants. Thus, our results support that overexpression of *IbNFU1* in sweetpotato plants increases the ascorbate recycling activity to detoxify ROS generated under salt stress by stimulating the ascorbate-mediated ROS scavenging (Figure 9) [33], [69], [74].

In addition, it was shown that there was no clear correlationship between salt tolerance of transgenic sweetpotato plants and copy number of the integrated gene, similar to the results reported by Gao et al. [2], in which the copy number of integrated *AtLOS5* gene ranged from 1 to 3 in transgenic plants exhibiting similar salt tolerance. Liu et al. [24] also indicated that the *IbP5CR*-overexpressing sweetpotato plants displayed different transgene integration patterns, but had similar salt tolerance.

In conclusion, we showed novel functions of the *IbNFU1* gene in regulation of proline accumulation, photosynthesis and ROS-scavenging system. Overexpression of *IbNFU1* significantly enhanced salt tolerance of the transgenic sweetpotato plants. It is suggested that the *IbNFU1* gene is involved in sweetpotato salt tolerance and enhances salt tolerance of the transgenic sweetpotato plants by regulating osmotic balance, protecting membrane integrity and photosynthesis and activating ROS scavenging system.
